# Redetermination of bis­(acetyl­acetonato-κ^2^ *O*,*O*′)(1,10-phenanthroline-κ^2^ *N*,*N*′)manganese(II)

**DOI:** 10.1107/S1600536813028614

**Published:** 2013-10-26

**Authors:** Stefan Suckert, Inke Jess, Christian Näther

**Affiliations:** a Institut für Anorganische Chemie, Christian-Albrechts-Universität Kiel, Max-Eyth-Strasse 2, 24118 Kiel, Germany

## Abstract

In the crystal structure of the title compound, [Mn(C_5_H_7_O_2_)_2_(C_12_H_8_N_2_)], the Mn^2+^ cation is coordinated by one bidentate 1,10-phenanthroline ligand and two acetyl­acetonate anions within a slightly distorted N_2_O_4_ octa­hedron. The asymmetric unit consists of one Mn^2+^ cation situated on a twofold rotation axis, one half of a 1,10-phenanthroline ligand and one acetyl­acetonate anion. In comparison with the previous determination based on visually estimated intensities recorded on precession photographs, the current redetermination with image-plate data reveals bond lengths and angles with much higher precision.

## Related literature

For the previous determination of the crystal structure, see: Stephens (1977 ▶).
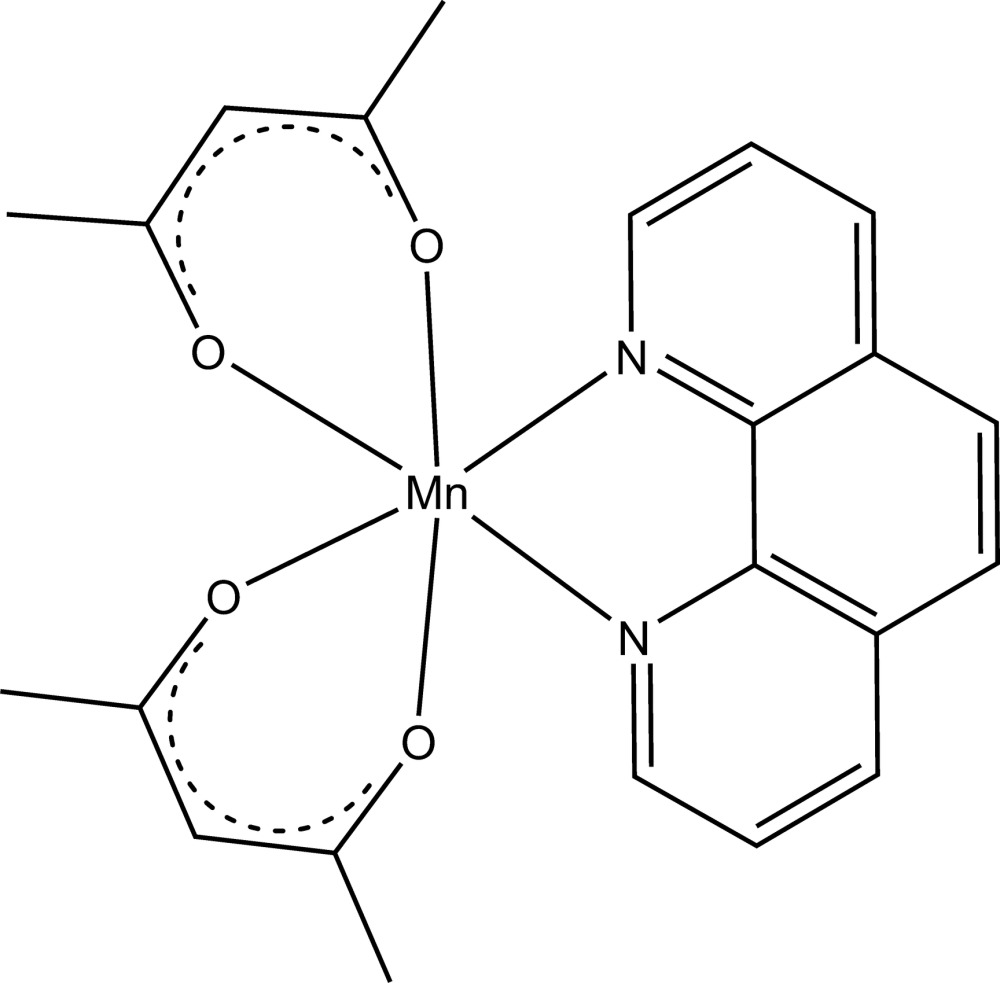


## Experimental

### 

#### Crystal data

[Mn(C_5_H_7_O_2_)_2_(C_12_H_8_N_2_)]
*M* *_r_* = 433.36
Orthorhombic, 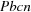

*a* = 15.8353 (7) Å
*b* = 10.2260 (4) Å
*c* = 12.6532 (4) Å
*V* = 2048.96 (14) Å^3^
*Z* = 4
Mo *K*α radiation
μ = 0.68 mm^−1^
*T* = 200 K
0.31 × 0.19 × 0.08 mm

#### Data collection

Stoe IPDS-2 diffractometer
14233 measured reflections
2007 independent reflections
1742 reflections with *I* > 2σ(*I*)
*R*_int_ = 0.086

#### Refinement

*R*[*F* ^2^ > 2σ(*F* ^2^)] = 0.047
*wR*(*F* ^2^) = 0.103
*S* = 1.16
2007 reflections
134 parameters
H-atom parameters constrained
Δρ_max_ = 0.26 e Å^−3^
Δρ_min_ = −0.26 e Å^−3^

### 

Data collection: *X-AREA* (Stoe & Cie, 2008 ▶); cell refinement: *X-AREA*; data reduction: *X-AREA*); program(s) used to solve structure: *SHELXS97* (Sheldrick, 2008 ▶); program(s) used to refine structure: *SHELXL97* (Sheldrick, 2008 ▶); molecular graphics: *XP* in *SHELXTL* (Sheldrick, 2008 ▶) and *DIAMOND* (Brandenburg, 2011 ▶); software used to prepare material for publication: *SHELXTL* and *publCIF* (Westrip, 2010 ▶).

## Supplementary Material

Crystal structure: contains datablock(s) I, global. DOI: 10.1107/S1600536813028614/wm2774sup1.cif

Structure factors: contains datablock(s) I. DOI: 10.1107/S1600536813028614/wm2774Isup2.hkl

Additional supplementary materials:  crystallographic information; 3D view; checkCIF report

## Figures and Tables

**Fig. 1.**
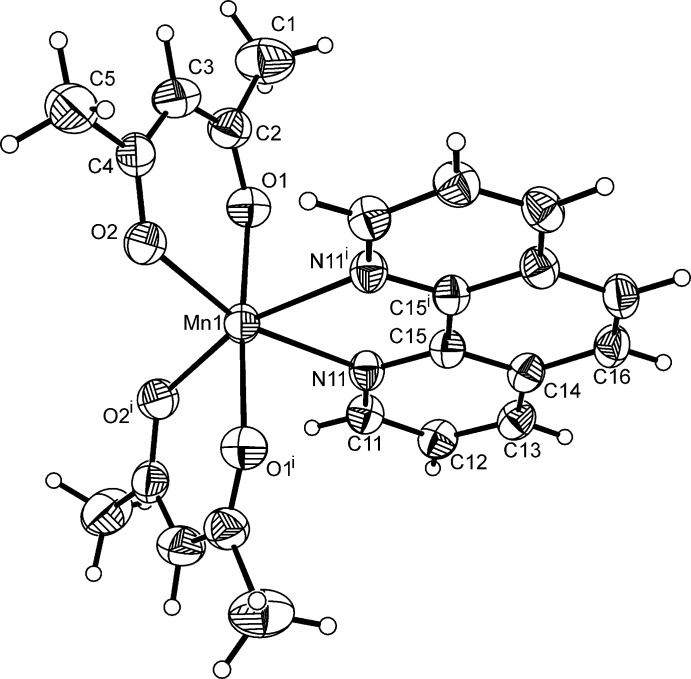
The molecular structure of the title compound with labelling and displacement ellipsoids drawn at the 50% probability level. [Symmetry code: i) -x+1,y,-z+3/2.]

**Fig. 2.**
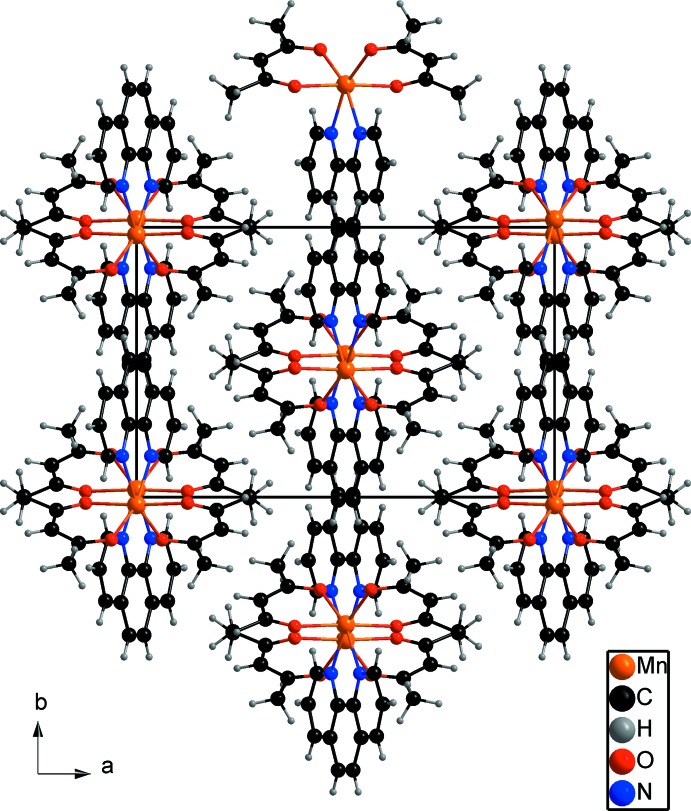
The crystal structure of the title compound in a projection along [001].

## supplementary crystallographic information

### 1. Comment

The title compound was serendipitously obtained within a project on the synthesis of manganese(III) coordination polymers containing cyanate anions and neutral N-donor co-ligands. Within this project manganese(III) acetylacetonate was reacted with 1,10-phenanthroline and potassium cyanate in acetonitrile leading to the formation of crystals of the title compound, the composition of which was determined by X-ray structure analysis. The crystal structure of this compound has already been reported by Stephens (1977) from visually estimated intensity data recorded on precession photographs. We decided to redetermine the structure on basis of image plate intensity data to achieve higher precision with respect to lattice parameters, atomic coordinates and resulting bond lengths and angles.

The manganese(II) cation is coordinated by two nitrogen atoms of the 1,10-phenanthroline ligand and four oxygen atoms of two symmetry-related acetylacetonate anions into discrete complexes that are located on twofold rotation axes (Fig. 1). The coordination polyhedron of the manganese(II) cation can be described as a slightly distorted MnN_4_O_2_ octahedron. Bond lengths and angles are comparable to those of the previous determination (Stephens, 1977), however with much higher precision, e.g. 2.2962 (18) Å for the Mn—N, and 2.1139 (16) and 2.1543 (16) Å for the two Mn—O bond lengths determined during the present redetermination *versus* 2.307 (5), 2.116 (5) and 2.152 (5) Å, respectively, of the previous determination.

Individual complex molecules are mainly held together by van der Waals forces. In the crystal structure, the discrete complexes are arranged in columns extending parallel to [001] (Fig. 2). Within these columns neighbouring complexes are related by centers of inversion.

### 2. Experimental

Manganese(III) 2,4-pentadionate and 1,10-phenanthroline were purchased from Alfa Aesar. Potassium cyanate was purchased from Fluka. The title compound was obtained by the reaction of 70.5 mg Mn(III) 2,4-pentadionate (0.20 mmol), 48.7 mg potassium cyanate (0.6 mmol) and 144.32 mg 1,10-phenanthroline (0.8 mmol) in 1.5 ml acetonitrile at RT in a closed 3 ml snap cap vial. After three days brown crystals of the title compound, mostly in the form of needles, were obtained by slow evaporation of the solvent.

### 3. Refinement

The aromatic hydrogen atoms were positioned with idealized geometry, methyl H atoms were allowed to rotate but not to tip, and were refined with *U*_eq_(H) = 1.2 *U*_eq_(C) for aromatic H atoms (1.5 for methyl H atoms) using a riding model with C—H = 0.95 Å (aromatic) and with C—H = 0.98 Å (methyl).

### Crystal data

**Table:**

[Mn(C_5_H_7_O_2_)_2_(C_12_H_8_N_2_)]	*F*(000) = 900
*M**_r_* = 433.36	*D*_x_ = 1.405 Mg m^−^^3^
Orthorhombic, *P**b**c**n*	Mo *K*α radiation, λ = 0.71073 Å
Hall symbol: -P 2n 2ab	Cell parameters from 17733 reflections
*a* = 15.8353 (7) Å	θ = 1.9–26.0°
*b* = 10.2260 (4) Å	µ = 0.68 mm^−^^1^
*c* = 12.6532 (4) Å	*T* = 200 K
*V* = 2048.96 (14) Å^3^	Plate, brown
*Z* = 4	0.31 × 0.19 × 0.08 mm

### Data collection

**Table:**

Stoe IPDS-2 diffractometer	1742 reflections with *I* > 2σ(*I*)
Radiation source: fine-focus sealed tube	*R*_int_ = 0.086
Graphite monochromator	θ_max_ = 26.0°, θ_min_ = 2.4°
ω scans	*h* = −19→19
14233 measured reflections	*k* = −10→12
2007 independent reflections	*l* = −14→15

### Refinement

**Table:**

Refinement on *F*^2^	Primary atom site location: structure-invariant direct methods
Least-squares matrix: full	Secondary atom site location: difference Fourier map
*R*[*F*^2^ > 2σ(*F*^2^)] = 0.047	Hydrogen site location: inferred from neighbouring sites
*wR*(*F*^2^) = 0.103	H-atom parameters constrained
*S* = 1.16	*w* = 1/[σ^2^(*F*_o_^2^) + (0.0406*P*)^2^ + 0.9028*P*] where *P* = (*F*_o_^2^ + 2*F*_c_^2^)/3
2007 reflections	(Δ/σ)_max_ < 0.001
134 parameters	Δρ_max_ = 0.26 e Å^−^^3^
0 restraints	Δρ_min_ = −0.26 e Å^−^^3^

### Special details

**Table:**

Geometry. All esds (except the esd in the dihedral angle between two l.s. planes) are estimated using the full covariance matrix. The cell esds are taken into account individually in the estimation of esds in distances, angles and torsion angles; correlations between esds in cell parameters are only used when they are defined by crystal symmetry. An approximate (isotropic) treatment of cell esds is used for estimating esds involving l.s. planes.
Refinement. Refinement of F^2^ against ALL reflections. The weighted R-factor wR and goodness of fit S are based on F^2^, conventional R-factors R are based on F, with F set to zero for negative F^2^. The threshold expression of F^2^ > 2sigma(F^2^) is used only for calculating R-factors(gt) etc. and is not relevant to the choice of reflections for refinement. R-factors based on F^2^ are statistically about twice as large as those based on F, and R- factors based on ALL data will be even larger.

### Fractional atomic coordinates and isotropic or equivalent isotropic displacement parameters (Å^2^)

**Table:**

	*x*	*y*	*z*	*U*_iso_*/*U*_eq_	
Mn1	0.5000	0.52826 (5)	0.7500	0.03271 (17)	
C1	0.23055 (17)	0.4974 (4)	0.8396 (3)	0.0658 (9)	
H1A	0.2197	0.4070	0.8179	0.099*	
H1B	0.1819	0.5521	0.8213	0.099*	
H1C	0.2398	0.5004	0.9162	0.099*	
C2	0.30819 (15)	0.5480 (3)	0.7834 (2)	0.0425 (6)	
C3	0.29703 (16)	0.6189 (3)	0.6895 (2)	0.0502 (7)	
H3	0.2406	0.6335	0.6668	0.060*	
C4	0.36143 (15)	0.6699 (2)	0.6268 (2)	0.0432 (6)	
C5	0.3379 (2)	0.7457 (3)	0.5282 (3)	0.0675 (9)	
H5A	0.3697	0.8279	0.5261	0.101*	
H5B	0.2772	0.7648	0.5291	0.101*	
H5C	0.3515	0.6935	0.4655	0.101*	
O1	0.37816 (10)	0.52168 (17)	0.82556 (13)	0.0406 (4)	
O2	0.43944 (10)	0.65765 (17)	0.64402 (14)	0.0435 (4)	
N11	0.53476 (11)	0.34743 (18)	0.84839 (14)	0.0328 (4)	
C11	0.56836 (14)	0.3481 (2)	0.94437 (18)	0.0362 (5)	
H11	0.5787	0.4300	0.9775	0.043*	
C12	0.58922 (14)	0.2340 (2)	0.99889 (19)	0.0386 (5)	
H12	0.6137	0.2388	1.0673	0.046*	
C13	0.57416 (14)	0.1155 (2)	0.95299 (18)	0.0377 (5)	
H13	0.5884	0.0371	0.9892	0.045*	
C14	0.53730 (13)	0.1099 (2)	0.85136 (18)	0.0341 (5)	
C15	0.51892 (13)	0.2298 (2)	0.80233 (17)	0.0315 (5)	
C16	0.51771 (14)	−0.0106 (2)	0.7983 (2)	0.0378 (5)	
H16	0.5300	−0.0915	0.8319	0.045*	

### Atomic displacement parameters (Å^2^)

**Table:**

	*U*^11^	*U*^22^	*U*^33^	*U*^12^	*U*^13^	*U*^23^
Mn1	0.0362 (3)	0.0284 (3)	0.0335 (3)	0.000	−0.00258 (19)	0.000
C1	0.0468 (15)	0.088 (3)	0.063 (2)	−0.0034 (15)	0.0073 (13)	0.0081 (17)
C2	0.0378 (12)	0.0447 (14)	0.0448 (14)	0.0015 (10)	0.0007 (10)	−0.0077 (11)
C3	0.0371 (12)	0.0531 (16)	0.0603 (18)	0.0019 (11)	−0.0077 (12)	0.0058 (13)
C4	0.0458 (13)	0.0320 (12)	0.0517 (15)	−0.0019 (10)	−0.0132 (11)	0.0024 (11)
C5	0.0617 (17)	0.0625 (19)	0.078 (2)	−0.0140 (15)	−0.0290 (16)	0.0291 (17)
O1	0.0387 (8)	0.0461 (10)	0.0370 (9)	0.0003 (7)	0.0000 (7)	−0.0026 (7)
O2	0.0406 (8)	0.0395 (9)	0.0504 (10)	−0.0021 (7)	−0.0056 (8)	0.0119 (8)
N11	0.0391 (9)	0.0296 (9)	0.0297 (10)	−0.0001 (7)	−0.0014 (7)	−0.0005 (8)
C11	0.0407 (11)	0.0359 (12)	0.0320 (12)	0.0009 (9)	−0.0012 (9)	−0.0016 (10)
C12	0.0422 (12)	0.0417 (13)	0.0320 (12)	0.0024 (10)	−0.0046 (9)	0.0014 (10)
C13	0.0411 (12)	0.0373 (13)	0.0348 (12)	0.0049 (10)	0.0005 (9)	0.0075 (10)
C14	0.0374 (11)	0.0330 (11)	0.0320 (12)	0.0028 (9)	0.0026 (9)	0.0021 (10)
C15	0.0346 (10)	0.0301 (11)	0.0297 (12)	0.0000 (8)	0.0016 (8)	0.0005 (9)
C16	0.0419 (12)	0.0289 (11)	0.0426 (14)	0.0024 (9)	0.0049 (10)	0.0051 (10)

### Geometric parameters (Å, º)

**Table:**

Mn1—O2^i^	2.1139 (16)	C5—H5A	0.9800
Mn1—O2	2.1139 (16)	C5—H5B	0.9800
Mn1—O1^i^	2.1543 (16)	C5—H5C	0.9800
Mn1—O1	2.1543 (16)	N11—C11	1.326 (3)
Mn1—N11^i^	2.2962 (18)	N11—C15	1.360 (3)
Mn1—N11	2.2962 (18)	C11—C12	1.395 (3)
C1—C2	1.512 (4)	C11—H11	0.9500
C1—H1A	0.9800	C12—C13	1.366 (3)
C1—H1B	0.9800	C12—H12	0.9500
C1—H1C	0.9800	C13—C14	1.413 (3)
C2—O1	1.259 (3)	C13—H13	0.9500
C2—C3	1.403 (4)	C14—C15	1.405 (3)
C3—C4	1.394 (4)	C14—C16	1.437 (3)
C3—H3	0.9500	C15—C15^i^	1.453 (4)
C4—O2	1.261 (3)	C16—C16^i^	1.344 (5)
C4—C5	1.515 (4)	C16—H16	0.9500
			
O2^i^—Mn1—O2	102.50 (10)	C4—C5—H5A	109.5
O2^i^—Mn1—O1^i^	83.96 (6)	C4—C5—H5B	109.5
O2—Mn1—O1^i^	98.30 (6)	H5A—C5—H5B	109.5
O2^i^—Mn1—O1	98.30 (6)	C4—C5—H5C	109.5
O2—Mn1—O1	83.96 (6)	H5A—C5—H5C	109.5
O1^i^—Mn1—O1	176.42 (10)	H5B—C5—H5C	109.5
O2^i^—Mn1—N11^i^	163.09 (7)	C2—O1—Mn1	126.41 (16)
O2—Mn1—N11^i^	92.95 (7)	C4—O2—Mn1	128.08 (16)
O1^i^—Mn1—N11^i^	87.07 (6)	C11—N11—C15	118.14 (19)
O1—Mn1—N11^i^	90.04 (7)	C11—N11—Mn1	126.04 (15)
O2^i^—Mn1—N11	92.95 (7)	C15—N11—Mn1	115.82 (14)
O2—Mn1—N11	163.09 (7)	N11—C11—C12	122.9 (2)
O1^i^—Mn1—N11	90.04 (6)	N11—C11—H11	118.5
O1—Mn1—N11	87.07 (6)	C12—C11—H11	118.5
N11^i^—Mn1—N11	72.71 (9)	C13—C12—C11	119.4 (2)
C2—C1—H1A	109.5	C13—C12—H12	120.3
C2—C1—H1B	109.5	C11—C12—H12	120.3
H1A—C1—H1B	109.5	C12—C13—C14	119.7 (2)
C2—C1—H1C	109.5	C12—C13—H13	120.2
H1A—C1—H1C	109.5	C14—C13—H13	120.2
H1B—C1—H1C	109.5	C15—C14—C13	116.9 (2)
O1—C2—C3	125.5 (2)	C15—C14—C16	119.8 (2)
O1—C2—C1	116.3 (2)	C13—C14—C16	123.3 (2)
C3—C2—C1	118.2 (2)	N11—C15—C14	123.0 (2)
C4—C3—C2	125.7 (2)	N11—C15—C15^i^	117.82 (12)
C4—C3—H3	117.1	C14—C15—C15^i^	119.19 (13)
C2—C3—H3	117.1	C16^i^—C16—C14	120.99 (13)
O2—C4—C3	125.5 (2)	C16^i^—C16—H16	119.5
O2—C4—C5	115.8 (2)	C14—C16—H16	119.5
C3—C4—C5	118.7 (2)		

Symmetry code: (i) −*x*+1, *y*, −*z*+3/2.
